# Engaging the public with antimicrobial resistance through social media videos—a content analysis study

**DOI:** 10.3389/fpubh.2026.1770727

**Published:** 2026-03-25

**Authors:** Fangyue Chen, Lakshya Soni, Kate Grailey, Simon Dryden, Amish Acharya, Ara Darzi

**Affiliations:** 1 Fleming Initiative, Institute of Global Health Innovation, Imperial College London, London, United Kingdom; 2 Institute of Global Health Innovation, Imperial College London, London, United Kingdom

**Keywords:** antimicrobial resistance, content analysis, engagement, online video, social media

## Abstract

**Introduction:**

YouTube, the dominant global video-sharing social media platform, has created new opportunities to provide regulated health content directly with the public, including on broader public health threats such as antimicrobial resistance (AMR). Little is known about the most effective strategy with which to engage the public with online AMR content.

**Method:**

This study comprehensively evaluated the top 200 viewed YouTube videos on AMR by extracting data on video characteristics, narratives and quality, and explored factors associated with viewer engagement through proxy analytics (views, likes and comments).

**Results:**

We found that content focused upon the mechanisms of AMR and antibiotics were most viewed, yet engaging videos do not necessarily convey high-quality information. Videos on internet media and non-medical channels were more popular.

**Discussion:**

The study calls for more strategic production of engaging videos on AMR. Global platforms should strive to facilitate audience in accessing reliable health information.

## 1 Introduction

The World Health Organization (WHO) has highlighted the importance of placing people, their needs and equitable access to health services at the center of addressing the global threat of Antimicrobial Resistance (AMR) (1). Primarily driven by antimicrobial overuse and misuse, the response to AMR depends upon understanding and targeting the complex interplay of human behaviors (1, 2). Greater emphasis has thus been placed on innovations around changing AMR-related behaviors among both the public and healthcare professionals (1).

At present, two-thirds of the global population use social media (3). Contrary to traditional media whose main role is information delivery, social media platforms enable the creation of online communities and facilitate multi-directional user interactions through various forms of communication, ranging from text-based (e.g., X) to visually-based (e.g., Instagram) (4). Widely leveraged by multi-sectoral stakeholders such as the governmental and commercial sectors to communicate with billions of users across geographical barriers, it has been of increasing interest to explore its potential in changing human behaviors, leading to both individual and collective improvement in health (5).

YouTube stands out as the dominant platform with a focus on video-based content (6). Presenting both auditory and visual information, videos can optimize the effectiveness of learning by maximizing the use of human cognitive infrastructure (7). They are advantageous in their ability to deliver consistent information across a large audience base, are especially receptive among those with low health literacy, and provides audience with control over the progress of learning (8).

While systematic reviews have indicated a degree of effectiveness on the use of social media in changing health-related behaviors such as smoking cessation, physical activity, protective behaviors during the COVID-19 pandemic, the overarching conclusion emphasized the importance of engaging the users with context-specific information that serves the unique needs of each community (9–12). For instance, McDonough et al. found an objective clinical improvement in individual’s physical activities levels after being shown a tailored social media video as compared with a general health education video (13). Moreover, when video interventions incorporate suitably designed messages, consideration of behavioral theories and audience engagement characteristics, evidence has shown that this has a positive impact on behavior-related outcomes such as knowledge and behavioral intent (14, 15).

Appropriate health communication on AMR is critical. The public have expressed incomplete understanding of AMR alongside misconceptions, while others find disaster framing messages such as “apocalypse” and “superbugs” meaningless and difficult to respond to with individual actions (16, 17). While AMR-related public campaigns have collectively shown positive impact on AMR-related behavioral outcomes, prior to October 2022, only one out of 41 studies describing AMR public health campaigns utilized social media as the main channel of communication (in this case social media hashtags) (18). The scale of impact from social media platforms like YouTube cannot be overlooked. In 2015, the UK Health Security Agency (then Public Health England) released a 30-s YouTube animation video showing dancing antibiotic pills singing the message “keep antibiotics working”. Although the video was released to accompany the main pledge-based Antibiotic Guardian campaign, it has garnered over 3 million views and directed the greatest number of unique visitors to the main campaign website (18, 19). Similar videos by Singapore Health Promotion Board were viewed nearly 1 million times (20). On the contrary, WHO has released several videos to raise awareness about AMR, yet they have been viewed fewer times, such as the video “What is antimicrobial resistance” with 7,700 views (21).

While the use of engagement analytics such as views cannot directly translate to impact on AMR-related behavioral change, the concept of “engagement” is considered critical to success when modeling the processes in which exposure to social media leads to behavioral change (22). Engagement in social media refers to any features or functions that allow users to interact, share and create content with their social networks (22). On YouTube, such engagement can range from passive content viewing to participation through single click-based interaction of “like,” or more actively through “commenting” (23).

Questions remain as to why certain AMR-related videos can generate greater engagement from the public. Previous studies analyzing general science-related YouTube content have found that certain video elements relate to its level of engagement: video structure such as its length and presence of an introduction, video topics, amount of information, visual quality, presenter characteristics and community integration (24, 25). Limited studies have examined AMR-related YouTube content and tended to focus on appraisal of video information quality, rather than factors that contribute toward their engagement (26, 27).

There is a need to better understand, thus tailor the social media messaging required to deliver public campaigns that appropriately tackle AMR-related human behaviors (18). The current study comprehensively reviewed AMR-related content on the largest video-based social media platform, YouTube, assessing video characteristics, video narratives, and the quality of information. Concurrently, we evaluated the relative importance of each element in generating social media engagement and found a greater level of engagement from videos hosted by internet media channels and videos with a narrative around the mechanism of AMR and antibiotics. It was anticipated that information from this study could provide a basis for the development of effective AMR-related public campaigns, leveraging the potential of video-based social media.

## 2 Materials and methods

We conducted the study online using publicly available data on YouTube (6), on which registered users can upload and openly share videos on their channels. Users can find video(s) according to their interests, most commonly by entering key related terms or phrases into the dedicated YouTube search bar. In addition, users can save selected videos into a playlist that can then be accessed later. Viewers can also become a subscriber of a specific channel to receive regular uploads. Each video has a unique identifiable code (Video ID). Each playlist has an ID that is sourced from the web address (also known as Uniform Resource Locator [URL]).

The YouTube platform highlights credible health-related content by using an information panel associated with the video content. Such authoritative health information has been identified by applying principles developed by an expert panel convened by the National Academy of Medicine and verified by the WHO, and the National Health Service (NHS) in the UK (28).

Ethical approval for this study was waived as all collected data were publicly available and anonymous.

### 2.1 Search strategy

A search on YouTube was conducted through a Google Chrome Incognito window (used to avoid previous searches from biasing the search results). The search term “antimicrobial resistance” was used. This term was selected as firstly, major international AMR campaigns such as the World AMR Awareness week utilizes this term, secondly, searching one term would simulate how individuals would typically search for content on YouTube. Thirdly, this term covers resistance of bacteria, viruses and fungi, without biasing toward a particular group of micro-organisms. Finally, searching one term would enable an adequate range of engagement metrics resulted from the selected sample of videos. In a systematic review by Osman et al., the median number of videos included in the analysis of health-related video content on YouTube is 94 (IQR 50.5–133) (29). The authors decided upon screening 200 videos, to give a comprehensive overview of YouTube AMR content within the resource constraints. The YouTube search algorithm can rank the results in four ways: relevance, upload date, view count and rating. Given that the article aims to evaluate the level of public engagement, of which view count is one of the important metrics, the top 200 videos ranked by view count from the search were collated and saved onto a dedicated YouTube playlist for screening for eligibility and data extraction.

### 2.2 Video screening

Two reviewers (FC and LS) screened all 200 videos in the playlist according to the eligibility criteria below. Disagreement between the two reviewers (FC and LS) were resolved by consensus.

#### 2.2.1 Inclusion criteria

Videos that discussed topics centered around AMR, which is defined as the phenomenon when bacteria, viruses, fungi and parasites no longer respond to antimicrobial medicines. Such topics include and are not limited to the following: mechanism of AMR and antimicrobial agents, actions against AMR, governmental legislations, AMR-related research, AMR-related patient experiences, expert interviews and talks, AMR surveillance.

#### 2.2.2 Exclusion criteria

When the video topic is not focused on AMR, but only briefly touches upon the subject. Non-English videos were excluded, given the difficulty in interpreting the video content in other languages by the authors.

### 2.3 Video feature extraction

#### 2.3.1 Development of the extraction framework

A conceptual framework was developed to facilitate comprehensive video feature extraction, informed by previous literature of YouTube content analysis (24, 25, 30, 31) and after discussion among authors (KG, AA, VS and FC). In summary, videos were examined in four broad aspects—channel information, video characteristics, video narrative and information quality (Supplementary Figure S4). All videos were watched in their entirety and relevant data were extracted manually by the reviewers (FC and LS).

#### 2.3.2 Channel information

Information on channel type, subscriber count and channel regions were extracted. Channel type was extracted by categories according to previous definitions (31) (Table 1). The latter two were extracted manually from the video channel page.

**Table 1:** Summary of video channel types.

Channel type (*n* = 163)	Definition	n (%)
Non-profit or medical organizations	Channels representing organizations such as hospitals, governmental organizations and universities	86 (52.8)
Educational by non-medical professionals	Educational or explanatory channels hosted by one or more individuals without healthcare professional qualifications	38 (23.3)
Educational by medical professionals	Educational or explanatory channels hosted by one or more individuals accredited with healthcare professional qualifications	13 (8.0)
Broadcasting agencies	Channels with associated broadcasting over television or radios	12 (7.4)
Internet media	Channels such as newsmagazine show or talk shows	7 (4.3)
Independent non-medical users	One or more individual(s) with no clear affiliation nor accreditation	5 (3.1)
Others	None of the above	2 (1.2)

#### 2.3.3 Video characteristics

Videos were categorized by style based on previous definitions (Table 2) (31). Informed by previous literature and author discussion, a list of specific video characteristics was assessed according to the definitions and signaling questions shown in Supplementary Table S9.

**Table 2:** Summary of video by style.

Video style (*n* = 163)	Definition	n (%)
Voice over visual	When there is narration over visual content	75 (46.0)
Presentation	When presenting to an audience	28 (17.2)
Hosted	One or more communicator(s) present information while other people are also part of the content	26 (16.0)
Vlog	Where the presenter(s) deliver content by talking directly to the camera	21 (12.9)
Text over visual	When there is text over visual content without narration	7 (4.3)
Interview	Where the person(s) delivering the content is being interviewed by person(s) off camera	6 (3.7)

#### 2.3.4 Video narratives

Video narratives on AMR encompass different AMR-related themes, such as mechanisms of AMR, mechanisms of action of antibiotics, actions against AMR and governmental policies/legislations related to AMR. In order to determine the AMR-related themes mentioned in each video, one reviewer (FC) viewed a sample of eligible videos and their corresponding transcripts (displayed for each video on the YouTube platform), and produced a list of cumulated themes until no further themes emerge (thematic saturation). Using this cumulative list, all eligible videos were then reviewed by two reviewers (FC and LS) to determine the AMR-related narrative themes mentioned in each video. Further themes that emerged and were outside of the pre-determined list were recorded qualitatively by the reviewers.

#### 2.3.5 Information quality

The quality of the information delivered in the video content was assessed by two reviewers (FC and LS) using DISCERN, a validated score-based tool (Supplementary Table S10). Although it was initially purposed for written health information, it has been extensively used to assess the quality of online video-based information (32). It is organized into three main sections: questions 1 to 8 assess the reliability of the publication, questions 9 to 15 focus on specific information regarding treatment choices and question 16 is the overall rating. Given that the videos mostly describe the situation of AMR in general, only sections 1 and 3 were utilized to assess the video quality. Section 2 was omitted given that it focuses on treatment choice evaluation. Each question contains a signaling question and is rated from 1 to 5 (1 being no, 3 being partially and 5 being yes). Each video was scored out of 45. The DISCERN scores were then classified into quality levels: Excellent quality (36–45); good quality (30–36); fair quality (23–29); poor quality (17–23); and very poor quality (9–17) (33).

#### 2.3.6 Data extraction process

Two reviewers (FC and LS) extracted the following datapoints manually, given that they involved decision-making around more than two options: categorization of videos by channel type and style, video narrative and quality assessment. When assessing the DISCERN score, given the high level of subjectivity in score assignment, scores that were within 1 point between the two reviewers were considered concordant, as per previous literature in Chan et al. (31). Disagreement between the two reviewers (FC and LS) were resolved by consensus.

One reviewer (FC) manually extracted data on the specific video characteristics, as they were considered more objective, requiring mostly a decision between two options.

### 2.4 Association between video features and engagement

Video characteristics, narratives, channel information and information quality were evaluated for their association with video engagement, defined as view count, views per day from the date of publication, like count and comment count. In addition, the association between the video channel types and information quality (DISCERN scores) were assessed.

YouTube video engagement data were extracted using YouTube Data Application program Interface (API) version 3, which allows automated, scaled-up extraction of YouTube data. Specifically, an API key was created over the Google Cloud Platform, restricted for YouTube Data API v3 (34). The playlist ID, sourced from the URL of the YouTube playlist containing the top 200 videos and the API key were then entered into pre-determined codes using R version 4.3.3 to extract the data. The following data were extracted:

YouTube video ID, video title, channel title, video publication date, video length, video view count, like count and comment count.

An additional variable, views per day was calculated from dividing view count from the time difference between publication date and the date that all data were extracted.

### 2.5 Statistical analysis

Categorical variables were summarized as number (%). These included video channel type, video style, AMR theme distribution and video characteristics. Numerical variables included the following: subscriber count, video duration, introduction duration, DISCERN score and engagement analytics (view count, views per day, like count and comment count). Shapiro-Wilks test was used to assess for normality. Parametric variables were expressed as mean and standard deviation; non-parametric variables were expressed as median and interquartile range. Complete case analysis was used for each engagement metrics to account for missing data.

The input variables, namely video characteristics, AMR-related themes and DISCERN score were evaluated for their association with outcome variables: view count, views per day, like count and comment count. For continuous input variables, Pearson’s correlation was used to assess association in the case of parametric outcome variables; spearman’s rank was used in the case of non-parametric outcome variables. The strength of association was assessed using correlation coefficient, and interpreted as described (Supplementary Table S11). For categorical variables with two categories, Mann–Whitney U test was utilized to compare the distribution of outcome variables between categories. For categorical variables with more than two categories, Kruskal-Wallis test was utilized to compare the distribution of outcome variables. If significant differences among the categories were identified, Dunn’s test was utilized to determine the specific group differences.

In addition, the correlation between the outcome variables (views per day, like count, comment count) were assessed against view count using the above method to assess for collinearity.

Interrater Reliability (IRR) between the two reviewers was assessed using Cohen’s Kappa (35). A Cohen’s Kappa of over 0.2 indicates fair interrater reliability, over 0.4 indicates moderate reliability, over 0.6 indicates substantial reliability, over 0.8 indicates almost perfect reliability (35).

All statistical analysis were performed using R version 4.3.3 in RStudio, using the following packages*: httr2, jsonlite, here, dplyr, ggplot2, rstatix, FSA, patchwork, corrplot, irr, MASS* (36–44).

## 3 Results

**Figure 1:**
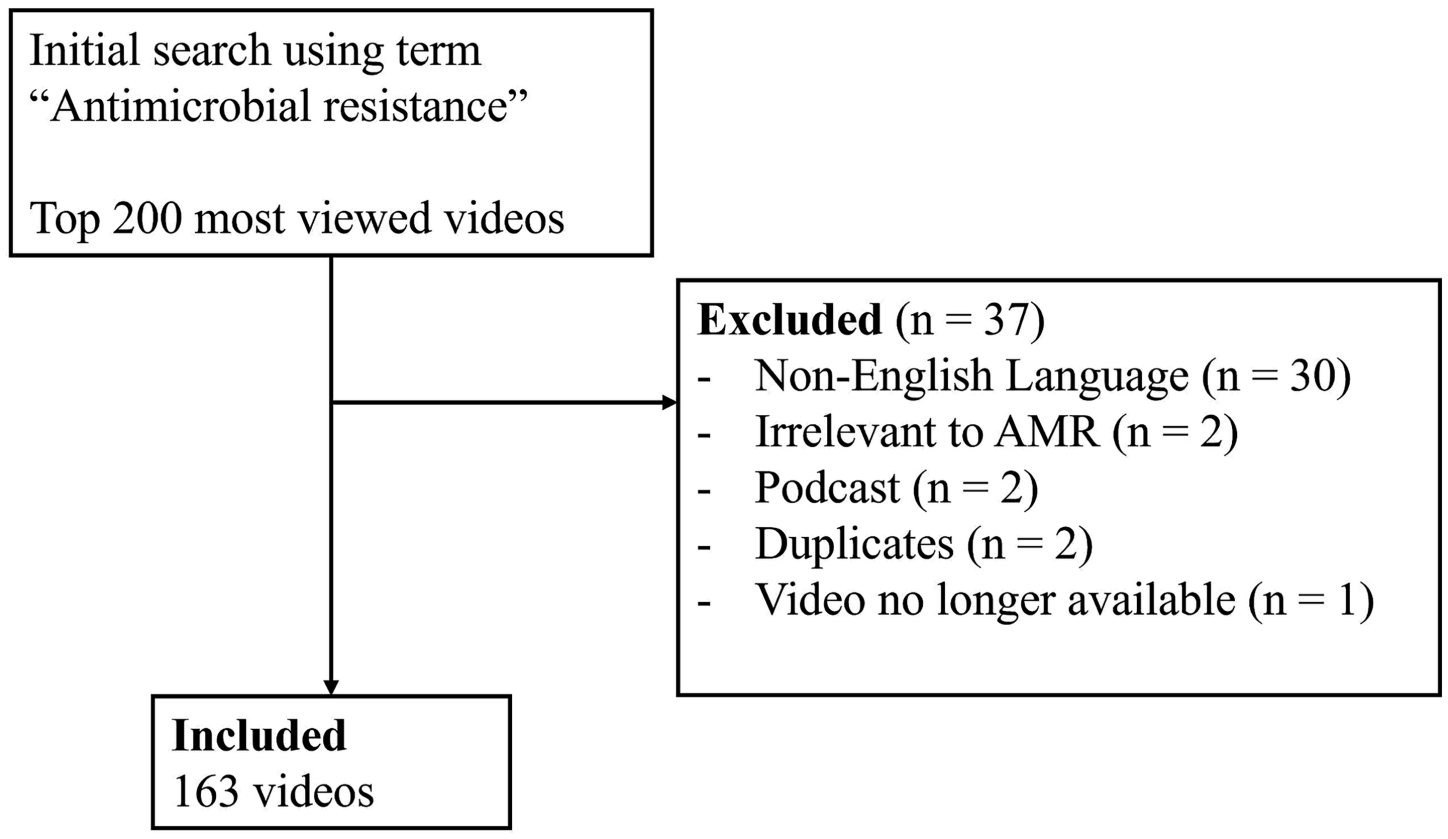
Video screening flowchart.

**Figure 2:**
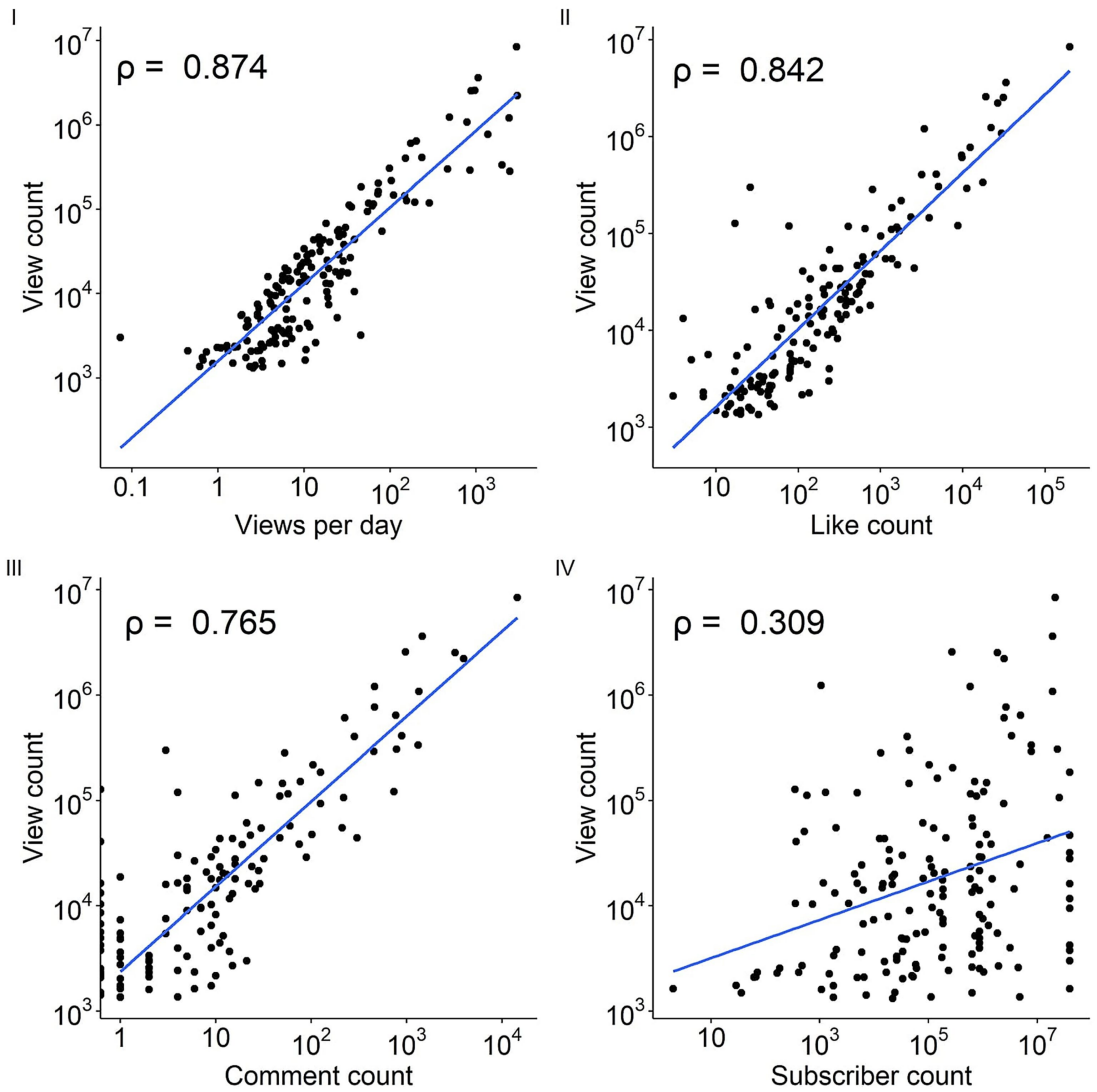
Correlation plot of video engagement analytics.

A YouTube search was conducted on 29th December 2023. The top 200 most viewed videos were screened against the eligibility criteria, resulting in 163 videos (Figure 1). The most popular AMR video generated over 8 million views, while the median view count is 14,006. The videos had a median duration of 4.7 min, ranging from 30s to over 2 h. The oldest video was published 4,734 days (nearly 13 years) ago (Table 3). The video view count correlated well with like count (*ρ* = 0.842), comment count (ρ = 0.765), views per day (ρ = 0.874), but less well with the subscriber count (ρ = 0.309; Figure 2).

**Table 3:** Summary of numerical variables, including video engagement analytics, video characteristics and quality of health information.

Numerical variables	Median (IQR)	Range (Min–Max)	Total
Views	14,006 (3419–47,095)	1,315–8,422,447	31,250,988
Views per day	10.0 (4.1–29.7)	0.07–3008.4	N/A
Likes	140 (43–602)	3–200,683	492,288
Comments	9 (1–29.3)	0–14,439	34,272
Subscribers	59,900 (5415–908,500)	2–39,600,000	218,768,533
Duration in min	4.7 (2.3–13.1)	0.5–137.8	2057.7
Days since publication	1,555 (657.5–2,357)	71–4,734	
DISCERN score	25 (22.3–29)	10.5–41	

### 3.1 Summary of video features

Non-profit or medical organizations constitutes the most common channel type (52.8%), followed by educational channels by non-medical professionals (23.3%) and educational channels by medical professionals (7.98%; Table 1). Fifty-one (31.3%) videos were from the United States, followed by 35 from United Kingdom (21.5%), 16 from India (9.82%), 14 from global institutions (8.60%). Of the 30 excluded videos that were not in English, 23 (76.7%) were from India, followed by two from Pakistan (6.67%; Supplementary Table S1).

Near half of the videos (46.0%) were of voice-over visual style, followed by presentations (17.2%; Table 2). Most videos contained an introduction to the topic (75.5%) and music (68.1%). Around 60% of the videos did not show the presenter’s accreditation. Only 12 of the 163 videos (7.4%) had an element of humor. Thirty-three out of 163 videos (22.2%) were highlighted by YouTube as “health-related content” (Table 4).

**Table 4:** Association between all investigated video characteristics and engagement analytics.

Categories	Signaling questions	Options	View count			Views per day		Comment count			Like count		
			n	Median (IQR)	*p*-value	Median (IQR)	*p*-value	n	Median (IQR)	*p*-value	n	Median (IQR)	*p*-value
Thumbnail	Is the thumbnail a screenshot during the video or specially designed?	Special feature	**73**	**20,822 (4007–115,694)**	**0.014**	**19.3 (5.0–72.3)**	**0.001**	**71**	**268 (49–1,477)**	**0.004**	**70**	**13 (3–77)**	**0.001**
		Screenshot during the video	90	9908 (2759–27,962)		6.7 (3.1–18.1)		78	92 (33–328)		66	5 (1–16)	
	Does the thumbnail contain texts?	Yes	98	15,650 (4206–84,557)	0.069	**14.3 (5.1–52.4)**	**0.004**	**91**	**229 (54–905)**	**0.004**	**83**	**11 (3–59)**	**0.015**
		No	65	8222 (2697–31,757)		6.6 (2.9–18.1)		58	79 (33–315)		53	5 (1–16)	
	Does the thumbnail contain channel logo?	Yes	43	15,048 (3899–41,350)	0.858	11.0 (5.8–33.4)	0.287	42	189 (61–516)	0.595	41	9 (2–47)	0.591
		No	120	13,328 (3287–51,619)		9.6 (3.6–28.5)		107	140 (35–607)		95	9 (1–29)	
Introduction	Does the video have an introduction?	Yes	123	14,781 (3803–49,035)	0.589	10.5 (4.0–30.0)	0.596	114	189 (44–646)	0.243	106	9 (2–43)	0.407
		No	40	9986 (2882–43,593)		8.1 (4.1–22.4)		35	94 (40–255)		30	6.5 (1–15)	
	Does the video introduction mention the video topic/aim?	Yes	99	15,908 (3899–54,798)	0.429	10.7 (4.2–31.2)	0.268	92	196 (45–677)	0.796	85	10 (2–47)	0.847
		No	25	12,332 (3043–38,415)		6.7 (2.9–26.1)		23	170 (45–580)		22	7 (3–27)	
	Does the video introduction include a question?	Yes	29	13,233 (2763–115,694)	0.728	10.7 (4.2–80.7)	0.436	27	136 (39–1,477)	0.789	25	11 (2–53)	0.631
		No	95	14,781 (3924–39,570)		10.3 (4.0–27.0)		88	189 (46–571)		82	9 (2–29)	
	Does the video start music immediately?	Yes	102	12,783 (3241–43,907)	0.479	9.0 (4.1–28.4)	0.798	90	136 (37–623)	0.903	83	8 (1–40)	0.481
		No	61	16,252 (3764–61,050)		11.0 (4.0–30.4)		59	188 (45–532)		53	10 (2–24)	
	Does the video start narration immediately?	Yes	53	16,457 (4454–93,715)	0.338	11.0 (4.2–54.7)	0.358	51	188 (40–443)	0.759	45	9 (2–47)	0.903
		No	104	13,620 (3351–43,534)		10.2 (4.1–28.0)		94	146 (44–646)		88	9 (1–29)	
	Does the video introduce the channel name immediately?	Yes	96	16,070 (4181–55,247)	0.102	10.6 (5.0–30.1)	0.163	**89**	**237 (78–656)**	**0.008**	**84**	**11 (2–50)**	**0.031**
		No	67	9601 (2488–44,167)		7.2 (3.0–29.5)		60	70 (27–331)		52	5 (1–17)	
	Length of introduction (s)	n	Median (IQR)		0.471								
		123	12 (7–27.3)										
Music	Does the video have music?	Yes	88	12,783 (3177–44,900)	0.91	8.6 (3.3–32.4)	0.862	78	113 (34–623)	0.446	71	5 (1–27)	0.148
		No	52	16,070 (3691–74,382)		11.1 (4.2–33.2)		49	188 (45–558)		44	11 (4–34)	
		Partly (during introduction and/or ending)	23	15,048 (3963–42,575)		10.8 (5.0–23.9)		22	270 (83–494)		21	10 (3–30)	
	Is the video music track named?	Yes	**28**	**33,494 (4992–132,961)**	**0.038**	**25.5 (6.8–80.0)**	**0.009**	26	393 (45–1,544)	0.083	26	14 (2–104)	0.093
		No	90	9431 (3018–27,685)		6.7 (3.4–23.3)		79	111 (31–376)		72	5 (1–17)	
	How fast is the music playing? * beats per minute	Slow (up to 76 bpm*)	30	15,288 (3324–52,233)	0.828	11.5 (3.5–37.0)	0.507	26	170 (37–788)	0.821	26	13 (1–58)	0.810
		Medium (76 to 120 bpm)	36	10,428 (3967–26,183)		8.6 (3.8–24.0)		30	117 (47–553)		23	5 (1–22)	
		Fast (> 120 bpm)	31	14,513 (2656–115,398)		10.7 (4.9–93.4)		30	113 (28–485)		27	5 (1–20)	
Animation	Does the video contain animations?	Yes	**81**	**16,394 (5487–111,868)**	**0.016**	12.2 (4.7–62.0)	0.054	72	216 (38–1,361)	0.332	64	8 (1–48)	0.725
		No	82	9524 (2632–29,827)		9.0 (3.5–20.0)		77	136 (45–379)		72	9 (2–22)	
	Does the animation follow a color scheme?	Yes	58	20,182 (7742–114,738)	0.335	18.2 (5.6–63.5)	0.528	54	235 (44–1,369)	0.886	46	10 (1–43)	0.809
		No	22	13,196 (5392–24,124)		8.9 (4.7–41.2)		18	218 (48–728)		17	5 (1–50)	
	Does the animation have a main character feature?	Yes	40	14,033 (5350–112,825)	0.68	14.9 (4.6–66.2)	0.658	35	135 (63–1,477)	0.484	31	5 (1–44)	0.661
		No	38	18,041 (5952–49,824)		9.9 (5.6–28.6)		33	241 (38–445)		29	9 (1–28)	
	Does the animation have sound effects	Yes	19	40,725 (9241–209,646)	0.24	19.8 (4.6–244.8)	0.493	15	114 (28–6,137)	0.798	14	8 (0–651)	0.851
		No	62	14,287 (4940–64,612)		11.0 (4.7–53.7)		57	229 (44–800)		50	8 (1–30)	
Presenter/interviewees	Is the presenter accreditation shown?	Yes	59	10,547 (3579–28,447)	0.134	9.9 (4.1–19.0)	0.191	57	140 (46–379)	0.264	51	7 (1–22)	0.254
		No	99	14,324 (3628–113,781)		10.6 (4.1–59.2)		87	229 (36–1,469)		80	11 (2–83)	
	Main narrator/presenter gender	Male	**62**	**12,262 (3394–36,529)**	**0.046**	**8.6 (3.2–27.2)**	**0.045**	59	114 (40–553)	0.32	**50**	**5 (1–18)**	**0.046**
		Female	83	16,394 (4817–112,793)		11.0 (4.9–63.0)		75	202 (47–1,365)		72	13 (3–58)	
		Both	4	3320 (2599–4,975)		3.9 (1.8–8.9)		4	141 (44–231)		4	5 (3–9)	
	Does the video have additional interviewees?	Yes	40	4396 (2334–22,257)	**0.009**	6.3 (3.3–15.4)	**0.050**	37	52 (27–239)	0.012	33	3 (1–15)	0.095
		No	**123**	**15,048 (4629–59,067)**		**10.9 (4.2–37.0)**		112	206 (46–654)		103	10 (2–40)	
	Does the video have expert interviewees?	Yes	37	5445 (2329–24,325)	**0.028**	6.5 (3.3–16.9)	0.113	34	77 (34–254)	0.073	30	5 (1–16)	0.333
		No	**126**	**14,647 (4293–56,471)**		10.8 (4.2–35.2)		115	202 (45–640)		106	10 (2–32)	
	Does the video have lay interviewees?	Yes	14	11,278 (2705–166,866)	0.964	8.3 (4.1–62.5)	0.710	13	52 (26–800)	0.324	13	2 (2–53)	0.902
		No	148	14,033 (3745–44,897)		10.4 (4.1–28.7)		135	188 (45–582)		122	9 (1–30)	
	Number of interviewees	n	Median (IQR)		0.5711								
		40	3 (1–5)										
Social interactions	Does the video encourage social interactions?	Yes	106	14,781 (4008–53,630)	0.257	10.8 (4.5–33.1)	0.177	97	190 (46–656)	0.251	91	9 (1–39)	0.251
		No	57	9446 (2697–43,362)		9.2 (3.3–24.5)		52	135 (34–409)		45	10 (2–28)	
	Does the channel owner reply to the top comments?	Yes	11	17,605 (3938–37,729)	0.412	24.4 (6.6–29.9)	0.947	10	265 (68–554)	0.741	11	12 (9–26)	0.741
		No	105	18,153 (4239–109,891)		11.2 (5.0–46.2)		104	238 (77–901)		105	11 (4–57)	
	Does the channel owner like the top comments?	Yes	13	18,040 (13,035 – 47,456)	0.976	11.2 (8.3–25.6)	0.287	13	331 (204–612)	0.287	13	16 (7–30)	0.287
		No	104	17,824 (3673–110,385)		11.4 (4.6–48.3)		102	205 (50–848)		104	11 (3–51)	
Visual quality	What is the visual quality of the video	> = 1080p	118	10,532 (3018–44,226)	0.182	9.7 (4.0–33.1)	0.984	107	136 (36–619)	0.555	97	9 (2–47)	0.555
		< 1080p	45	19,806 (4950–54,961)		10.0 (4.5–24.5)		42	206 (56–496)		39	9 (1–23)	
Humor	Does the video include elements of humor?	Yes	**12**	**115,547 (34,642 – 412,501)**	**0.001**	**42.4 (18.6–579.8)**	**0.002**	**11**	**2,575 (317–14,411)**	**0.004**	**9**	**301 (23–768)**	**0.004**
		No	151	12,332 (3133–40,889)		9.0 (3.8–25.8)		138	136 (39–496)		127	8 (1–25)	
YouTube Health	Is the video highlighted as “health-related content” by YouTube?	Yes	33	14,006 (3957–38,119)	0.895	10.0 (5.0–16.9)	0.926	32	138 (74–256)	0.875	25	7 (4–15)	0.875
		No	130	13,647 (3241–53,630)		10.1 (3.7–35.0)		117	152 (34–656)		111	9 (1–40)	

Bold values indicate video characteristics that were significantly associated with the indicated engagement metric.

Most videos discussed general actions against AMR (71.2%), followed by mechanism of AMR (70.6%), and the description of antimicrobial over-use and over-prescription (69.3%; Table 5). Other themes included infection control (28 videos; 17.2%), one-health/multi-sectoral actions (23 videos; 14.1%), and AMR surveillance (8 videos; 4.91%). On reviewing video narratives, one reviewer (FC) reviewed 20 videos until thematic saturation.

**Table 5:** Frequency of each AMR-related topic and association with engagement analytics.

Is the AMR topic mentioned?		View count				Views per day		Like count				Comment count			
		n	%	Median (IQR)	*p*-value	Median (IQR)	*p*-value	n	%	Median (IQR)	*p*-value	n	%	Median (IQR)	*p*-value
Actions against AMR	Y	116	71.2	11,129 (3445–43,393)	0.365	8.8 (4.0–25.5)	0.161	104	69.8	140 (38–515)	0.320	95	69.9	9 (2–27)	0.544
	N	47	28.8	16,231 (3532–102,792)		18.0 (4.1–45.8)		45	30.2	190 (45–1,010)		41	30.1	10 (1–125)	
Mechanisms of AMR	Y	115	70.6	**18,153 (5065–81,643)**	**<0.001**	**12.2 (4.6–42.0)**	**0.002**	106	71.1	**249 (48–752)**	**0.001**	99	72.8	**11 (3–55)**	**<0.001**
	N	48	29.4	4231.5 (2334–14,482)		6.0 (2.8–14.0)		43	28.9	78 (25–200)		37	27.2	2 (0–10)	
Antimicrobial usage and over-prescription	Y	113	69.3	12,332 (3470–43,362)	0.728	9.9 (4.0–28.5)	0.603	102	68.5	161 (43–551)	0.864	95	69.9	9 (2–27)	0.968
	N	50	30.7	15,146 (3324–66,216)		10.5 (4.1–37.3)		47	31.5	135 (37–759)		41	30.1	8 (1–102)	
Severity of AMR (epidemiology; consequences without)	Y	97	59.5	13,422 (3043–43,484)	0.444	9.9 (3.7–25.6)	0.401	88	59.1	140 (42–530)	0.596	82	60.3	9 (2–28)	0.899
	N	66	40.5	14,554 (3755–85,549)		10.6 (4.3–46.0)		61	40.9	190 (45–753)		54	39.7	8 (1–55)	
Agricultural and environmental factors linked with AMR	Y	77	47.2	11,710 (3298–44,285)	0.552	6.7 (3.7–34.6)	0.296	68	45.6	135 (42–616)	0.849	62	45.6	11 (2–47)	0.233
	N	86	52.8	15,640 (3970–49,824)		10.6 (4.2–28.9)		81	54.4	188 (44–558)		74	54.4	7 (1–21)	
Antibiotics and mechanisms of action	Y	55	33.7	**28,025 (7743–147,772)**	**<0.001**	**19.3 (6.4–92.0)**	**<0.001**	50	33.6	**479 (136–2379)**	**<0.001**	47	34.6	**16 (8–175)**	**<0.001**
	N	108	66.3	8781 (2583–28,047)		6.8 (3.1–19.9)		99	66.4	84 (33–328)		89	65.4	4 (1–16)	
Governmental policies/Legislations	Y	50	30.7	8459 (2468–26,411)	**0.031**	5.7 (2.9–15.3)	**0.011**	45	30.2	98 (27–415)	0.068	41	30.1	7 (1–28)	0.262
	N	113	69.3	**16,231 (4007–67,938)**		**11.2 (4.7–38.6)**		104	69.8	206 (46–658)		95	69.9	9 (2–38)	
Drug development in antibiotics and AMR	Y	48	29.4	17,031 (3966–79,877)	0.149	14.0 (4.7–42.0)	0.219	46	30.9	240 (45–788)	0.128	44	32.4	**13 (4–79)**	**0.042**
	N	115	70.6	10,547 (3260–37,375)		9.0 (4.0–28.1)		103	69.1	111 (41–479)		92	67.6	6 (1–21)	
Novel research in AMR (e.g., phage therapy, novel diagnostics)	Y	39	23.9	3764 (2635–49,060)	0.091	6.7 (3.0–35.7)	0.346	37	24.8	107 (45–561)	0.702	35	25.7	10 (2–42)	0.648
	N	124	76.1	15,478 (4904–46,915)		10.6 (4.4–29.4)		112	75.2	189 (43–605)		101	74.3	8 (1–28)	
Interviews with AMR-related experts	Y	36	22.1	6069 (2284–28,318)	**0.043**	6.3 (3.0–15.1)	0.054	34	22.8	77 (33–304)	0.061	31	22.8	4 (1–19)	0.208
	N	127	77.9	**14,513 (4008–59,067)**		10.7 (4.3–37.0)		115	77.2	202 (46–701)		105	77.2	10 (2–47)	
AMR- related laboratory experiments	Y	33	20.2	23,522 (3631–117,913)	0.184	**19.0 (6.2–56.5)**	**0.038**	32	21.5	**369 (51–1913)**	**0.045**	29	21.3	**21 (5–283)**	**0.006**
	N	130	79.8	13,328 (3394–40,074)		8.9 (3.7–25.9)		117	78.5	135 (43–415)		107	78.7	7 (1–22)	
AMR patient experiences/cases	Y	27	16.6	15,908 (3320–50,848)	0.917	9.2 (3.9–30.3)	0.998	26	17.4	136 (45–643)	0.922	24	17.6	9 (2–59)	0.788
	N	136	83.4	13,328 (3445–45,078)		10.2 (4.1–29.4)		123	82.6	152 (44–560)		112	82.4	9 (1–28)	

Bold values indicate video characteristics that were significantly associated with the indicated engagement metric.

Videos had a median DISCERN score of 25 (out of 45), indicating “fair” quality. Most videos had clear aims, had achieved their aims, and were relevant for their audience. Most videos had showed poorly the sources and dates of the information from which they were based, and often did not discuss areas of uncertainty, knowledge gaps or differences in opinions (Supplementary Figure S1).

### 3.2 Association between video features and levels of engagement

#### 3.2.1 Videos hosted by internet media channels were most engaging

**Figure 3:**
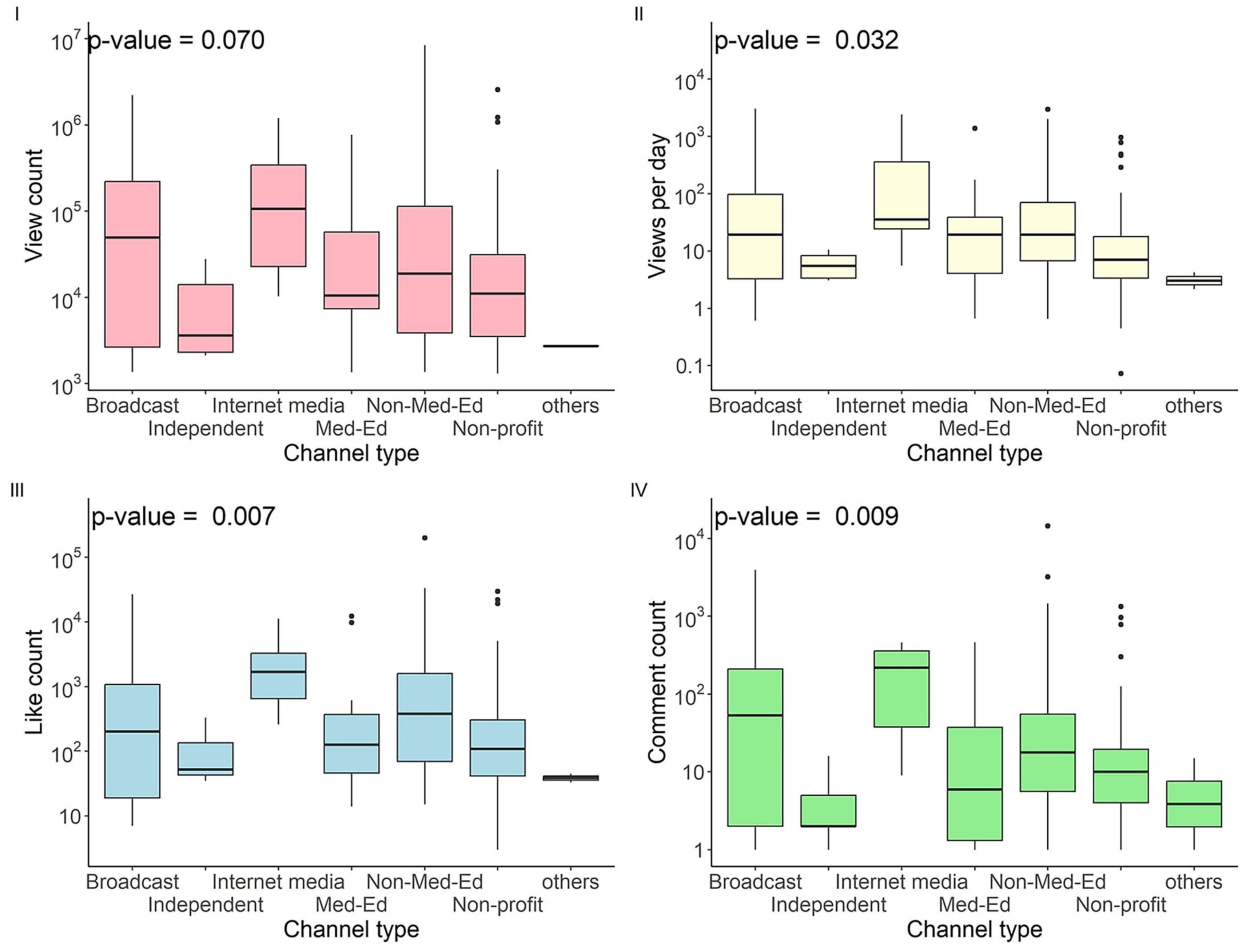
Channel type and video engagement (view count, views per day, like count, comment count).

Videos on different channel types differ significantly in views per day, like count and comment count (Figure 3). Internet media channels showed the greatest level of engagement (see Supplementary Tables S2–S4). As compared with independent channels, those videos had significantly more views per day (*p* = 0.014), like count (*p* = 0.011), comment count (*p* = 0.012). Comparing with non-profit or medical organizations, such videos have more views per day (*p* = 0.006), like count (*p* = 0.001), comment count (*p* = 0.001). Internet media also shows greater like count (*p* = 0.015) and comment count (*p* = 0.015) compared with channels by healthcare professionals. Educational channels run by non-healthcare professionals shows greater engagement than non-profit or medical organizations, seen in views per day (*p* = 0.009), like count (*p* = 0.008) and comment count (0.009).

#### 3.2.2 Specially designed thumbnails, animation content and humour were important in engagement

The analysis of key video characteristics and their association with video engagement is shown in Table 4. Having specially designed thumbnails was associated with significantly greater number of views (*p* = 0.014), views per day (*p* = 0.001), likes (*p* = 0.004) and comments (*p* = 0.001). Having texts in the thumbnail was associated with greater views per day (*p* = 0.004), likes (*p* = 0.004) and comment counts (*p* = 0.015). Animation content was associated with a greater number of views (*p* = 0.016). Including elements of humor appeared very important in generating engagement (*p* = 0.001 for views; *p* = 0.002 for views per day; *p* = 0.004 for like count; *p* = 0.025 for comment count), yet only 12 out of 163 AMR videos included this. No association was found between videos styles and the level of engagement (Supplementary Figure S2). When breaking down significant characteristics by channel types, we found that the lowest number of videos from channels hosted by non-profit/medical organizations used specially designed thumbnails (26.7% of videos), as compared to 71.1% of non-medical educational channels, 71.4% of internet media channels, and 61.5% of medical educational channels (Supplementary Table S5).

Notably, having additional interviewees in the video was associated with an adverse association with engagement (*p* = 0.009 for views; *p* = 0.050 for views per day), especially when in the presence of expert interviewees (*p* = 0.028 for views).

Including an introduction, background music, high visual quality and channel owner’s engagement activities with viewers were not associated with greater engagement. Videos that were accredited as health-related content was not associated with greater engagement.

#### 3.2.3 Videos describing mechanisms of AMR and antibiotics showed greater engagement

Table 5 shows the number of videos in which each of the 12 AMR themes have been mentioned, and their association with engagement analytics. Videos describing antibiotics and mechanisms of action, and mechanisms of antimicrobial resistance were associated with significantly greater level of engagement, including greater number of views (*p* < 0.001; *p* > 0.001), views per day (*p* < 0.001; *p* = 0.002), like count (*p* < 0.001; *p* = 0.001) and comment count (*p* < 0.001; *p* < 0.001), respectively. “AMR-related laboratory experiments” was associated with greater number of views per day (*p* = 0.038), like count (*p* = 0.045) and comment count (*p* = 0.006). Conversely, governmental actions/legislations, and interview with AMR experts were associated with significantly less engagement, including fewer views (*p* = 0.031; *p* = 0.043), fewer views per day (*p* = 0.011; *p* = 0.054) and marginally fewer likes (*p* = 0.068; *p* = 0.061), respectively.

#### 3.2.4 Levels of engagement did not correlate with information quality using DISCERN

**Figure 4:**
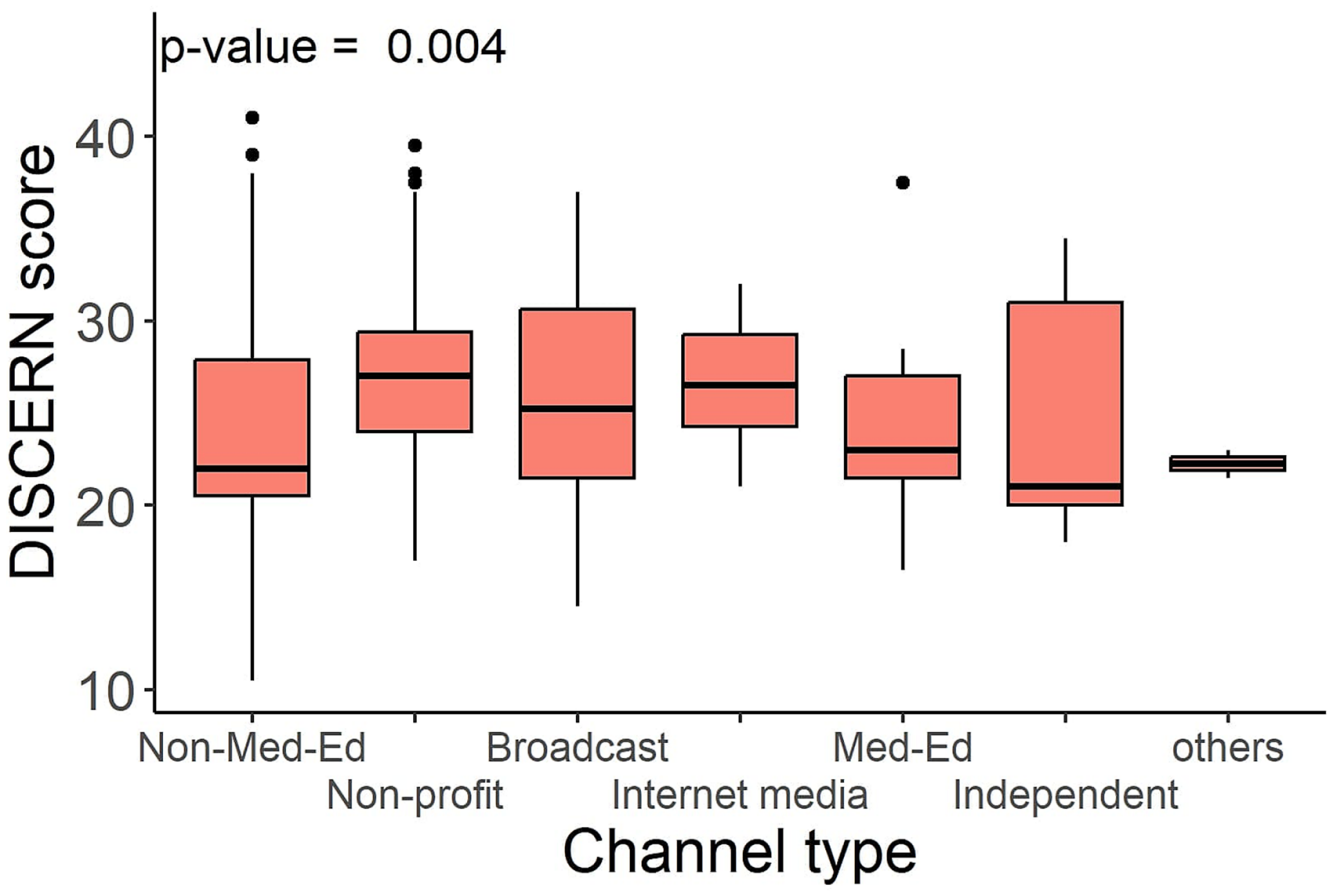
DISCERN score by channel type.

The correlation coefficients for numerical variables are collectively shown in Supplementary Figure S3. There was no significant correlation between engagement analytics and DISCERN score. Significant difference in DISCERN score was found in videos from different channel types (Figure 4). Specifically, videos from non-profit or medical organizations were of better quality than educational videos from non-medical professionals (*p* < 0.001; Supplementary Tables S6, S7).

### 3.3 Inter-rater reliability

The interrater reliability between the two reviewers (FC and LS) is shown in Supplementary Table S8. Most procedures showed moderate to substantial reliability, although DISCERN score showed a lower reliability (Cohen’s Kappa 0.32).

## 4 Discussion

This content analysis study highlighted the importance of the strategic composition of social media online videos to engage the public and convey information about AMR. Notably, we found differential public engagement in videos incorporating different AMR-related themes: videos that discussed mechanisms of AMR and antibiotics appeared more engaging; individuals were more ambivalent toward the mention of the AMR severity and patient experiences; videos detailing governmental legislations and actions appeared less engaging. While most AMR-related videos were hosted on channels by non-profit or medical organizations, videos on internet media channels were more engaging. General video characteristics such as a well-designed thumbnail and humor can also generate greater engagement. Such differential engagement indicates that audience on YouTube are particularly drawn to certain AMR-related themes, video characteristics and channel types.

Strategic health communication has been key in effectively conveying important health concerns to the public, such as climate change, COVID-19 related behaviors and vaccination program participation (45–47). Frameworks guiding effective communication exist, such as the WHO general strategic communication framework centered around six principles (actionable, credible, relevant, timely, understandable and accessible) and more recently, a social media–based public health campaign framework (48, 49). Most however, have overlooked the interactive nature of social media. Goering et al. applied the Uses and Gratifications (U&G) framework to explore individuals’ motifs for seeking health information on YouTube (50). The framework focuses on the motives for media use, audience activity and the result of the use such as satisfaction. They found that individuals seek health-related information on YouTube as a means for convenience, online community for social support, exciting entertainment, and passing time. As a result, individuals gain satisfaction through a sense of empowerment, or a perceived level of control and competence over their healthcare (50). Similarly, Khan et al. found that individuals were motivated to consume YouTube for relaxation and entertainment (23). Applying the U&G framework to our study findings, mechanisms of AMR and antibiotics are knowledge-based topics that the public may prefer to seek for, and through gaining a better understanding of the subject, they gain personal satisfaction and empowerment. Moreover, internet media channels were found to be more engaging, as they may provide a greater level of entertainment as compared to educational channels and non-profit organization and medical channels. This also applies to video characteristics including well-design thumbnails, elements of humor and incorporation of animations within the videos. Notably, a distinctively lower proportion of videos hosted on channels by non-profit and medical organizations were found to have specially designed thumbnails compared to other channel types, which is a particular area identified for improvement.

With regards to specific AMR-related themes, our study demonstrated that audiences appeared more enthused to learn about the mechanisms behind AMR. This is consistent with systematic reviews summarizing public’s knowledge and awareness of AMR, which have indicated an incomplete understanding of AMR among the public, and many would learn more from internet and social networks, the second largest information sources behind doctors (17). Contrarily, individuals appeared ambivalent toward the description of AMR severity. This is similarly found in communications around climate change, a global issue that share features with AMR in which individuals who act for their immediate benefit and convenience can negatively impact the collective society (51). Threat-based content tends to render audience powerless in identifying actionable steps, thus fuelling public indifference (47). However, during the COVID-19 pandemic, threat-based communication appeared to be more effective, especially when encouraging individuals to carry out specific actions such as vaccination (52). This may suggest that when threat is more immediately felt, coupled with clear actionable mitigation strategies, audience may better engage with health-related conversations to initiate behavioral change. AMR, often described as the “silent pandemic,” may not have generated as much immediate threat to the public, such that they are engaged to elicit behavioral change (53). While patient experiences often form an important part of public health and advocacy, this study found that individuals appeared ambivalent toward videos that included patient experiences. This could be explained by the general public’s relative unfamiliarity with the concept of AMR and the preference to be educated of its core mechanism. Moreover, AMR patient stories can often be emotionally-intensive, and a previous content analysis of patient stories on breast cancer found that stories that were less emotionally intensive tended to be more engaging (54).

Our work showed that videos mentioning governmental policies/legislations and those that included interviews with AMR-related experts appeared less engaging. Moreover, videos hosted by non-profit or medical organizations were collectively viewed less, despite the information being of higher quality, as shown by the DISCERN scores. While the subscriber count of such channels be a potential confounding factor unaccounted for, the relatively weak correlation between subscriber count and view count (Figure 2) indicated additional factors accounting for such differences in engagement among channel types. Similar findings were reported in previous content analysis of YouTube videos on public health threats such as Ebola and Zika (55, 56). Such lack of engagement could be explained by the Messenger effect, as part of the MINDSPACE behavioral science framework summarizing nine powerful influencers of human behaviors (57). Specifically, individuals are heavily influenced by those who communicate information. The distrust in official bodies is a known phenomenon, as seen surrounding vaccination programs, climate change initiatives and effective COVID-19 interventions (58). This can negatively impact how the public engages with the communicated information, thus explain our study findings. This contrasts against the rising popularity of social media influencers (SMIs), individuals who have generated a large network of followers through the release of their digital content, and are generally considered more connected with their followers and authentic in their fields (59). This could be due to the fact that demographic and behavioral similarities between the messenger and the recipient can improve the effectiveness of the communication (57). Nevertheless, how the credibility of the messenger impacts audience’s transition from engagement to behavioral change is an avenue for further exploration.

The study provides insight into how YouTube can be more effectively utilized to generate AMR-related behavior change. While audiences globally use YouTube, they may not voluntarily look for AMR-related content, and as a result important messages such as specific call to actions against AMR can be overlooked. Engagement can be improved by strategically incorporating video elements that align with audience’s inclination for empowerment and entertainment, through collaborating with internet media and educational channels by non-medical professionals, and by partnering with SMIs who already have a large network of followers (59). Videos can be shown more strategically in context, such as around the patient’s health-seeking episode, so that patients can better associate AMR with their own health and daily actions.

Finally, our study accentuates the importance of governing the quality of health information across all channel types, given individuals better engage with non-health related channels which tend to show information of poorer quality. YouTube has shown initiative in governing health-related information, through accrediting content as “health-related content,” and the “health content shelf”, collating accredited health information (28). Nevertheless, our study found no association between as the accreditation of “Health-related content” and engagement. While the public can be made aware of how to access credible health-related content on YouTube, this process can be automated by embedding credibility in YouTube’s recommendation algorithm. Currently, the algorithm aims to direct users toward the most relevant content to them, thereby maximizing user satisfaction. As such, the algorithm ranks videos based on engagement analytics such as click-through rate (the likelihood of clicking on the video after seeing it), watch time, videos seen per channel and YouTube-directed satisfaction surveys (60). The recommending algorithm should incorporate evaluation of the quality of content such as to prioritize the reach of good quality health information.

### 4.1 Limitations

This study has several limitations. Firstly, only videos delivered in English language were analyzed. Twenty-three of the 30 excluded non-English videos originated from India, the country with the greatest number of YouTube audience (3). Future studies could therefore explore how languages and countries of origin can impact the video content and engagement related to AMR.

Secondly, in assessing the information quality of each video, DISCERN tool was used, which was not specifically designed for videos. Moreover, the inter-rater reliability using such tool was low at *κ* = 0.32. This calls for the development of more robust, validated, video-specific tools to better assess information quality.

The study investigated video engagement from openly available YouTube data, rather than analyzing specific engagement analytics available to the channel owners themselves, such as watch time, audience demographics and locations (61). This information can provide more comprehensive and granular information on how the audience interact with a particular video. This data was not incorporated due to the requirement to collaborate with individual channel owners, but this provides an avenue for future more in-depth evaluation. In addition, information on how channel owners have attempted to enhance video engagement, through methods such as embedding the videos in other channels and paid advertising can be immensely useful, but will require collaboration with channel owners to collect such information.

Furthermore, the study did not assess engagement from openly available text data from video comments, which can provide greater information on the audience’s reaction to individual videos, and may shed light on how videos can impact individual’s health beliefs and AMR-related health behaviors. Moreover, it remains uncertain who the audience of the AMR videos were, and what their intentions were when searching for and consuming online videos of interest.

### 4.2 Opportunities for future work

There are several areas for future exploration. Firstly, collaborating with channel owners to collect more granular engagement metrics would provide more comprehensive insights into how a global audience engages with AMR-related videos, and explore for any country-specific or demographic-specific differences. Moreover, the thematic analysis of qualitative data within viewers comments could also provide insights into how videos impacted their health beliefs and health-related behaviors surrounding AMR. Finally, it remains unknown whether engagement with an online video can translate to objective impact on individual’s health-related behaviors. This may call for the implementation of such videos in a more controlled setting, so that change in an individual’s knowledge, behaviors and ultimately health outcomes can be objectively evaluated. Such work has been undertaken by the CE4AMR network, which has run projects generating participatory video content and evaluated the impact of such videos on knowledge, attitudes and behaviors toward AMR (62). Whether incorporating characteristics associated with greater engagement can translate to meaningful impact on individual’s behavioral determinants relating to AMR is yet to be explored.

## 5 Conclusion

Given the growing importance of AMR, and the potential of social media platforms such as YouTube to increase public engagement in these complex and challenging topics, it is essential that we create videos that are designed to maximize impact, engagement and collective benefit. This study provides insight into the design of more effective public health campaigns against antimicrobial resistance (AMR) that can be utilized by creators, policy makers and public health professionals. Our data demonstrates that online video content should be created with the audience in mind. Given the global reach of YouTube, and its potential to be a trusted source of public health information, it is imperative that both creators and policy makers utilize this in the best way possible, creating engaging content that provides reliable health information, and inspiring health-related behavior change.

## Data Availability

The original contributions presented in the study are included in the article/Supplementary material, further inquiries can be directed to the corresponding author.
