# Supplementary material for: Engaging the public with antimicrobial resistance through social media videos—a content analysis study
Source: Front Public Health. 2026 Mar 25;14:1770727. doi: 10.3389/fpubh.2026.1770727 (PMC13057502; doi:10.3389/fpubh.2026.1770727)
Supplement: Supplementary file 1 [file Supplementary_file_1.docx]

Supplementary Material

**Supplementary Table S1.** Number of videos by location

| **Included (n=163) n (%)** | | **Excluded non-English (n=30) n (%)** | |
| --- | --- | --- | --- |
| United States | 51 (31.3) | India | 23 (76.7) |
| United Kingdom | 35 (21.5) | Pakistan | 2 (6.7) |
| India | 16 (9.8) | Yemen | 1 (3.3) |
| Global | 14 (8.6) | Mexico | 1 (3.3) |
| Australia | 8 (4.9) | Ghana | 1 (3.3) |
| Germany | 6 (3.7) | Bangladesh | 1 (3.3 |
| Italy | 6 (3.7) | Uncertain | 1 (3.3) |
| Sweden | 5 (3.1) |  |  |
| Canada | 4 (2.5) |  |  |
| Europe | 4 (2.5) |  |  |
| Uncertain | 4 (2.5) |  |  |
| Nordic | 3 (1.8) |  |  |
| Ireland | 1 (0.6) |  |  |
| Kenya | 1 (0.6) |  |  |
| Nigeria | 1 (0.6) |  |  |
| Singapore | 1 (0.6) |  |  |
| Switzerland | 1 (0.6) |  |  |
| Kenya/UK | 1 (0.6) |  |  |


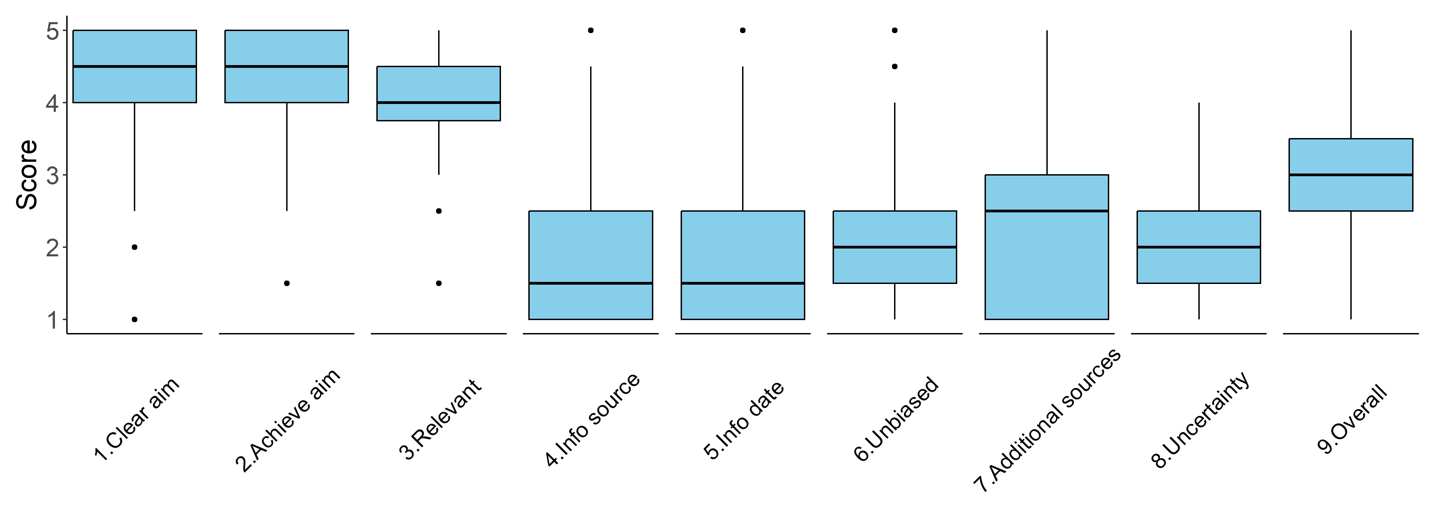
**Supplementary Figure S1.** DISCERN scores by item

**Supplementary Table S2.** Dunn’s multiple comparison tests for the association between channel types and view count per day since publication. P-value <0.05 is considered significant

|  |  |  |
| --- | --- | --- |
| **Comparison** | **Z** | **P-value** |
| Broadcast - Independent | 1.30 | 0.194 |
| Broadcast - Internet media | -1.56 | 0.119 |
| **Independent - Internet media** | **-2.45** | **0.014** |
| Broadcast – Med-Ed | 0.098 | 0.922 |
| Independent – Med-Ed | -1.24 | 0.216 |
| Internet media – Med-Ed | 1.67 | 0.096 |
| Broadcast - Non-med-Ed | -0.50 | 0.616 |
| Independent - Non-med-Ed | -1.80 | 0.072 |
| Internet media - Non-med-Ed | 1.40 | 0.162 |
| Med-Ed - Non-med-Ed | -0.64 | 0.523 |
| Broadcast – Non-profit | 1.11 | 0.269 |
| Independent – Non-profit | -0.76 | 0.447 |
| **Internet media – Non-profit** | **2.75** | **0.006** |
| Med-Ed – Non-profit | 1.01 | 0.310 |
| **Non-Med-Ed – Non-profit** | **2.60** | **0.009** |
| Broadcast - others | 1.57 | 0.117 |
| Independent - others | 0.61 | 0.545 |
| **Internet media - others** | **2.42** | **0.016** |
| Med-Ed - others | 1.52 | 0.127 |
| Non-Med-Ed - others | 1.88 | 0.060 |
| Non-profit - others | 1.20 | 0.231 |

**Supplementary Table S3.** Dunn’s multiple comparison tests for the association between channel types and like count. P-value <0.05 is considered significant

| **Comparison** | **Z** | **P-value** |
| --- | --- | --- |
| Broadcast - Independent | 0.71 | 0.476 |
| **Broadcast - Internet media** | **-2.27** | **0.023** |
| **Independent - Internet media** | **-2.53** | **0.011** |
| Broadcast - Med-Ed | 0.11 | 0.915 |
| Independent - Med-Ed | -0.65 | 0.518 |
| **Internet media - Med-Ed** | **2.43** | **0.015** |
| Broadcast - Non-Med-Ed | -1.04 | 0.296 |
| Independent - Non-Med-Ed | -1.56 | 0.119 |
| Internet media - Non-Med-Ed | 1.77 | 0.076 |
| Med-Ed - Non-Med-Ed | -1.25 | 0.213 |
| Broadcast - Non-profit | 0.55 | 0.581 |
| Independent - Non-profit | -0.45 | 0.656 |
| **Internet media - Non-profit** | **3.23** | **0.001** |
| Med-Ed - Non-profit | 0.45 | 0.654 |
| **Non-Med-Ed - Non-profit** | **2.64** | **0.008** |
| Broadcast - others | 1.15 | 0.252 |
| Independent - others | 0.59 | 0.552 |
| **Internet media - others** | **2.47** | **0.014** |
| Med-Ed - others | 1.10 | 0.270 |
| Non-Med-Ed - others | 1.71 | 0.088 |
| Non-profit - others | 0.98 | 0.327 |

**Supplementary Table S4.** Dunn’s multiple comparison tests for the association between channel types and comment count. P-value <0.05 is considered significant

| **Comparison** | **Z** | **P-value** |
| --- | --- | --- |
| Broadcast – Independent | 1.44 | 0.149 |
| Broadcast – Internet media | -1.37 | 0.169 |
| **Independent – Internet media** | **-2.51** | **0.012** |
| Broadcast – Med-Ed | 1.14 | 0.256 |
| Independent – Med-Ed | -0.55 | 0.586 |
| **Internet media – Med-Ed** | **2.43** | **0.015** |
| Broadcast – Non-Med-Ed | 0.17 | 0.863 |
| Independent – Non-Med-Ed | -1.53 | 0.126 |
| Internet media – Non-Med-Ed | 1.79 | 0.073 |
| Med-Ed – Non-Med-Ed | -1.27 | 0.206 |
| Broadcast – Non-profit | 1.76 | 0.078 |
| Independent – Non-profit | -0.41 | 0.681 |
| **Internet media – Non-profit** | **3.21** | **0.001** |
| Med-Ed – Non-profit | 0.32 | 0.751 |
| **Non-Med-Ed – Non-profit** | **2.60** | **0.009** |
| Broadcast – others | 0.79 | 0.427 |
| Independent – others | -0.21 | 0.835 |
| Internet media – others | 1.61 | 0.107 |
| Med-Ed – others | 0.16 | 0.876 |
| Non-Med-Ed – others | 0.76 | 0.445 |
| Non-profit – others | 0.02 | 0.982 |


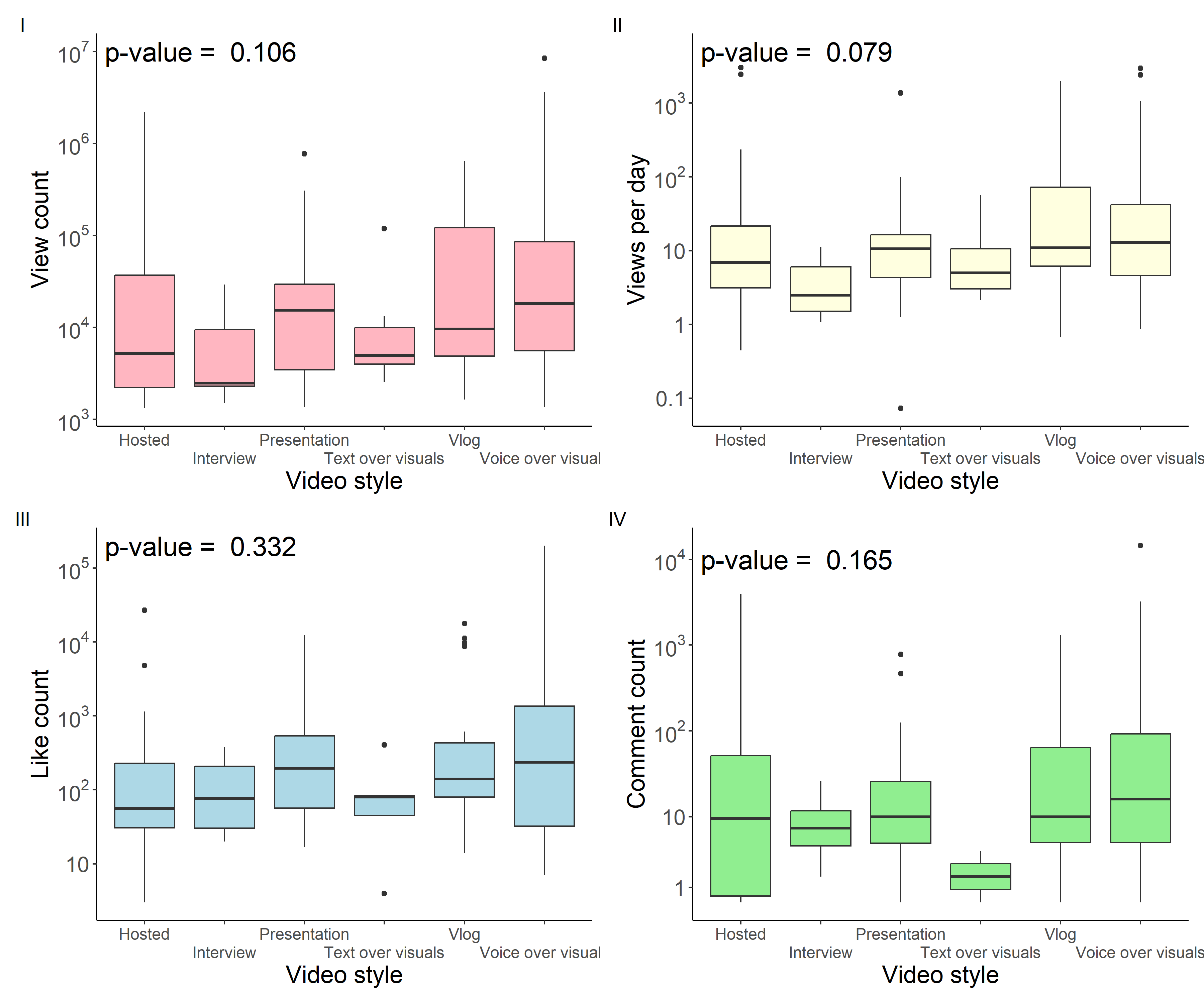


**Supplementary Figure S2.** Video style and engagement

| **Channel type** | **Special Thumbnail**  **n (%)** | **Animation**  **n (%)** | **Humour**  **n (%)** |
| --- | --- | --- | --- |
| Broadcasting agencies | 7 (58.3) | 3 (25.0) | 0 (0.0) |
| Education – non-medical professionals | 27 (71.1) | 25 (65.8) | 3 (7.9) |
| Education – medical professionals | 8 (61.5) | 3 (23.1) | 1 (7.7) |
| Independent non-medical users | 2 (40.0) | 1 (20.0) | 0 (0.0) |
| Internet media | 5 (71.4) | 4 (57.1) | 1 (14.3) |
| Non-profit / medical organisations | 23 (26.7) | 45 (52.3) | 7 (8.1) |
| Others | 1 (50.0) | 0 (0.0) | 0 (0.0) |

**Supplementary Table S5.** Video characteristics that were significantly associated with greater engagement and the breakdown by channel types.

| **Channel types** | **n** | **Median (IQR)** |
| --- | --- | --- |
| Broadcasting agencies | 12 | 25.3 (21.5 – 30.6) |
| Education by non-medical professionals (e.g. science education or explanatory media) | 38 | 22 (20.5 – 27.9) |
| Educational by medical professionals | 13 | 23 (21.5 – 27) |
| Independent non-medical users (e.g. vloggers with no obvious affiliations) | 5 | 21 (20 – 31) |
| Internet media (e.g. newsmagazine show or talk shows) | 7 | 26.5 (24.3 – 29.3) |
| Non-profit or medical organizations (e.g. hospitals, government organizations, universities) | 86 | 27 (24 – 29.4) |
| Others | 2 | 22.3 (21.9 – 22.6) |

**Supplementary Table S6.** DISCERN score by channel types


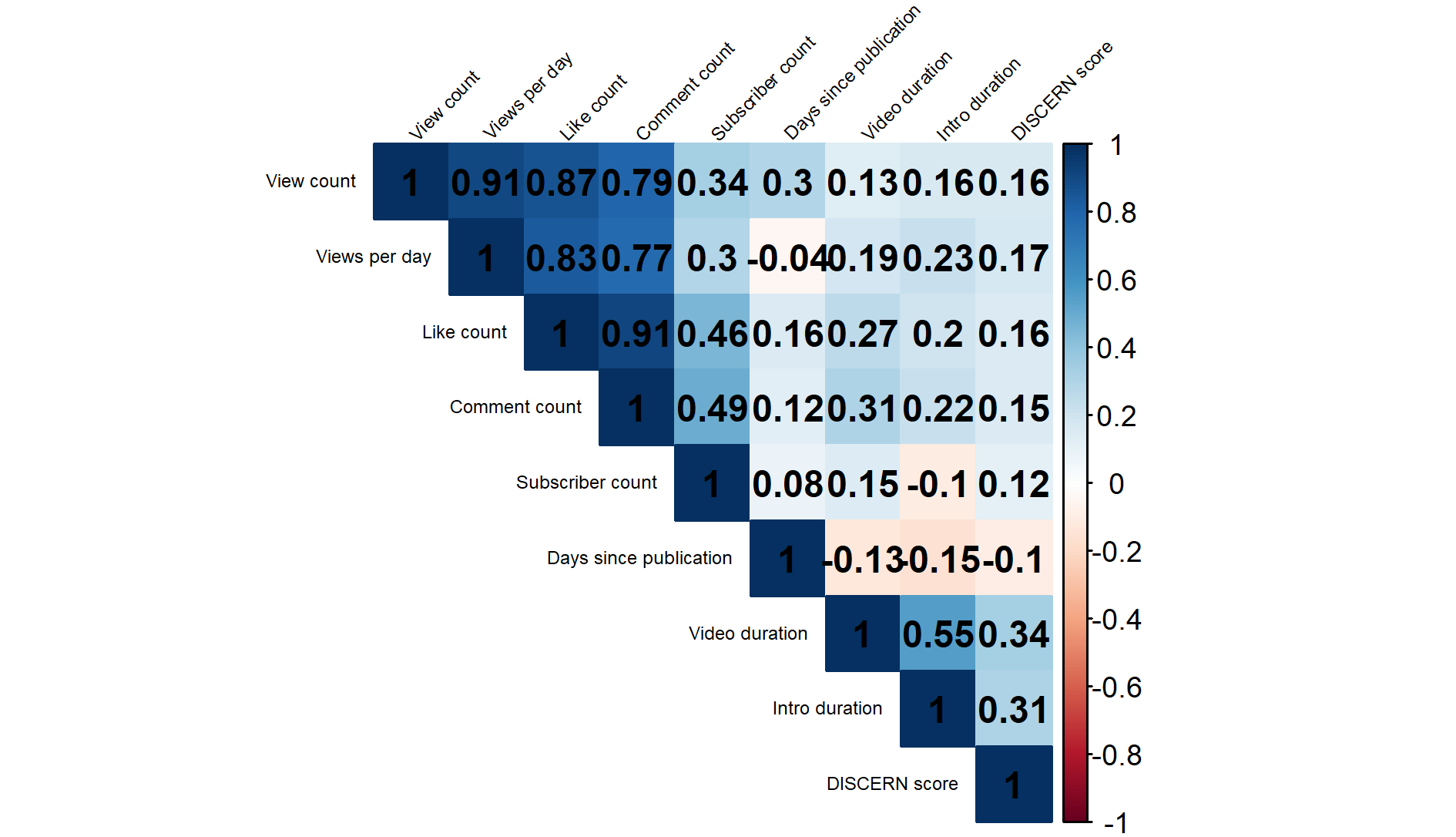


**Supplementary Figure S3.** Correlation matrix for numerical variables. Spearman’s correlation coefficient values are shown

**Supplementary Table S7.** Dunn’s multiple comparison tests for the association between channel types and DISCERN score. P-value <0.05 is considered significant

| Comparison | Z | P-value |
| --- | --- | --- |
| Broadcast - Independent | 0.509 | 0.611 |
| Broadcast - Internet media | -0.480 | 0.631 |
| Independent - Internet media | -0.853 | 0.394 |
| Broadcast - Med_Ed | 0.878 | 0.380 |
| Independent - Med_Ed | 0.153 | 0.878 |
| Internet media - Med_Ed | 1.237 | 0.216 |
| Broadcast - Non-med_Ed | 1.256 | 0.209 |
| Independent - Non-med_Ed | 0.304 | 0.761 |
| Internet media - Non-med_Ed | 1.566 | 0.117 |
| Med_Ed - Non-med_Ed | 0.200 | 0.842 |
| Broadcast - Non_profit | -1.071 | 0.284 |
| Independent - Non_profit | -1.307 | 0.191 |
| Internet media - Non_profit | -0.259 | 0.796 |
| **Med_Ed - Non_profit** | **-2.291** | **0.022** |
| **Non-med_Ed - Non_profit** | **-3.829** | **<0.001** |
| Broadcast - others | 1.071 | 0.284 |
| Independent - others | 0.654 | 0.513 |
| Internet media - others | 1.305 | 0.192 |
| Med_Ed - others | 0.614 | 0.539 |
| Non-med_Ed - others | 0.555 | 0.579 |
| Non_profit - others | 1.605 | 0.108 |

**Supplementary Table S8.** Inter-rater reliability in Cohen’s Kappa

| **Procedure** | **Cohen’s Kappa** | **Interrater reliability** |
| --- | --- | --- |
| Video screening | 0.93 | Almost perfect |
| Channel types | 0.67 | Substantial reliability |
| Video styles | 0.55 | Moderate reliability |
| Video topics | 0.56 | Moderate reliability |
| DISCERN | 0.32 | Fair reliability |


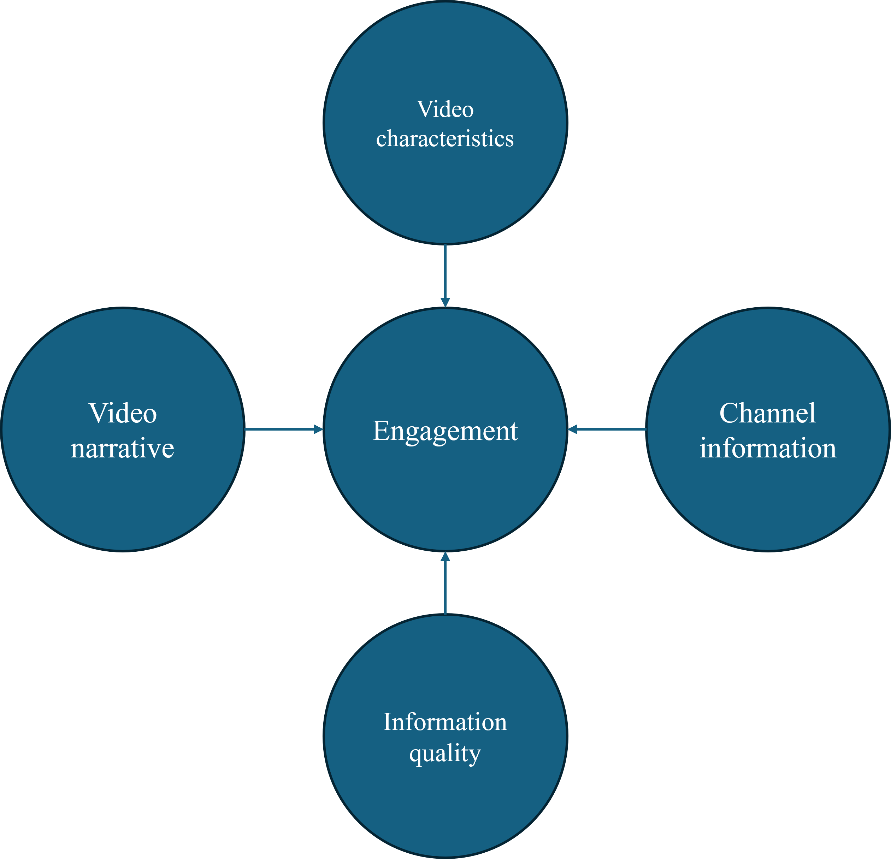


**Supplementary Figure S4.** Conceptual framework for video feature extraction

**Supplementary Table S9.** Video characteristics, definitions and options

| **Category** | **Definition** | **Signaling questions** | **Options** |
| --- | --- | --- | --- |
| **Thumbnail** | A still image that serves as the preview of the video. It can be specially designed or a screenshot of the video content. | Is the thumbnail a screenshot during the video or specially designed? | - Special feature - Screenshot during the video |
|  |  | Does the thumbnail contain texts? | - Yes - No |
|  |  | Does the thumbnail contain channel logo? | - Yes - No |
| Introduction | An initial segment of a video that is clearly separated from the main content of the video, usually by changes of music, a clear pause, a transition. | Does the video have an introduction? | - Yes - No |
|  |  | Does the video introduction include the video topic/aim? | - Yes - No |
|  |  | Does the video introduction include a question? | - Yes - No |
|  |  | Does the video start music immediately? | - Yes - No |
|  |  | Does the video start narration immediately? | - Yes - No |
|  |  | Does the video introduce the channel name immediately? | - Yes - No |
|  |  | Length of introduction | Time in seconds |
| Background Music | The sound that is laid over the visual content of the video and is not meant to have the audience’s focus, but often plays a role in setting the atmosphere and expressing emotions in videos. | Does the video have background music? | - Yes - No - Partly (during introduction and/or ending) |
|  |  | Is the music track mentioned? | - Yes - No |
|  |  | How fast is the music playing, defined by the tempo?  (If the rhythm changes, what’s the main tempo) | - Slow: up to 76 bpm* - Medium: 76 to 120 bpm - Fast: > 120 bpm |
| Animation | A technique used in motion pictures or video production, produced frame by frame, in which inanimate objects, such as cartoon drawings or puppets, appear to move of their own accord.  Colour scheme: a particular combination of colours that is used throughout the video.  Main character feature: a central character that repeatedly occur in the animation.  Sound effects: Sounds other than speech or music that are added to make it seem more exciting or real | Does the video contain animations? | - Yes - No - Partly |
|  |  | Does the animation follow a colour scheme? | - Yes - No |
|  |  | Does the animation have a main character feature? | - Yes - No |
|  |  | Does the animation have sound effects | - Yes - No |
| Presenter/  Narrator/  interviewees details | Presenter/narrator: The characters providing a background narration or speaking during the video.  Interviewees: individuals that are being interviewed  Lay: a person not trained in AMR. Typically, a patient or relative.  Expert: a professional individual in a field associated with AMR. | Is the presenter accreditation shown? | - Yes - No |
|  |  | Main narrator/presenter gender | - Male - Female - Other |
|  |  | Does the video have additional interviewees? | - Yes - No |
|  |  | Does the video have expert interviewees? | - Yes - No |
|  |  | Does the video have lay interviewees? | - Yes - No |
|  |  | The number of Interviewees | Number |
| Social interactions with the audience | Features on the YouTube platform that indicate elements of social interaction with the audience. Examples include content within the video that encourages like, comment and subscription, channel owner liking a comment or replying to a comment.  The interactions with the top comment of every video are chosen to standardize the level of interaction by the channel owner. | Does the video encourage social interactions? | - Yes - No |
|  |  | Does the channel owner like the top comments? | - Yes - No |
|  |  | Does the channel owner reply to the top comments? | - Yes - No |
| Visual quality | The resolution, clarity and overall visual appearance of a video  1080 resolution is considered full high-definition video | What is the visual quality of the video? | A visual quality indicator   - >= 1080p - < 1080p |
| Humor | Video content that is amusing or comical | Does the video include elements of humor? | - Yes - No |
| YouTube health information | An information panel provided by YouTube to indicate health-related content and its sources. | Is the video highlighted as “health-related content” by YouTube? | - Yes - No |

*bpm: beats per min

**Supplementary Table S10.** The DISCERN instrument items

| **Items** | **Abbreviation** |
| --- | --- |
| 1. Are the aims clear? | Clear aim |
| 2. Does it achieve its aims? | Achieve aim |
| 3. Is it relevant? | Relevant |
| 4. Is it clear what sources of information were used to compile the publication (other than the author or producer)? | Info source |
| 5. Is it clear when the information used or reported in the publication was produced? | Info date |
| 6. Is it balanced and unbiased? | Unbiased |
| 7. Does it provide details of additional sources of support and information? | Additional sources |
| 8. Does it refer to areas of uncertainty | Uncertainty |
| 9. Overall quality | Overall |

**Supplementary Table S11.** Correlation coefficient and interpretation of strength of correlation

| **Correlation Coefficient (ρ)** | **Interpretation** |
| --- | --- |
| 0.00 – 0.10 | Negligible correlation |
| 0.10 – 0.39 | Weak correlation |
| 0.40 – 0.69 | Moderate correlation |
| 0.70 – 0.89 | Strong correlation |
| 0.90 – 1.00 | Very strong correlation |
